# Using Molecular Markers to Help Predict Who Will Fail after Radical Prostatectomy

**DOI:** 10.1155/2011/290160

**Published:** 2011-04-14

**Authors:** Gregory P. Swanson, David Quinn

**Affiliations:** ^1^Departments of Radiation Oncology, Radiology, and Urology, University of Texas Health Science Center at San Antonio, 7703 Floyd Curl Drive MC 7889, San Antonio, TX 78229, USA; ^2^University of Southern California Norris Comprehensive Cancer Center, 1441 Eastlake Avenue Suite 3440, Los Angeles, CA 90033, USA

## Abstract

Recent phase III trial data clearly demonstrate that adjuvant therapy can reduce recurrence and increase survival after prostatectomy for prostate cancer. There is great interest in being able to accurately predict who is at risk of failure to avoid treating those who may not benefit. The standard markers consisting of prostate specific antigen (PSA), Gleason score, and pathological stage are not very specific, so there is an unmet need for other markers to aid in prognostic stratification. Numerous studies have been conducted with various markers and more recently gene signatures, but it is unclear whether any of them are really useful. We conducted a comprehensive review of the literature to determine the current status of molecular markers in predicting outcome after radical prostatectomy.

## 1. Introduction

Prostate specific antigen (PSA), stage (either clinical or pathological), and Gleason score are firmly established as prognostic indicators in prostate cancer. Individually and collectively, they predict for failure after radiation and surgery. The predictive value has been increased with more detailed information such as the addition of the detailed pathological findings of extraprostatic extension (EPE), positive margins, seminal vesicle involvement, and lymph node involvement. Various combinations of factors have been combined into tables, formulas, neural networks, and nomograms. While they are important tools in trying to predict failure, they are limited by the predictive ability of the factors themselves. For example, from a nomogram, a patient with a Gleason 7 (3 + 4) cancer with extraprostatic extension and positive margins, negative lymph nodes, negative seminal vesicles, and preoperative PSA of 8.2 ng/mL is predicted to have a 10-year recurrence rate of 20% [1]. Telling a patient that he has a 1 out of 5 chance of failing may or may not be reassuring. The absolute precision would be able to tell a patient whether he will fail (100%) or not (0%). The ultimate goal is to try to determine who *will* fail, not who *may* fail. The only way to try to approach that goal is with more precise markers than we currently have. One area of major promise in this regard is the greater individual cancer data that can be obtained from molecular markers.

We already have seen an example of the benefit of a marker in prostate cancer. The addition of the biological marker PSA offers more precise information over stage and Gleason score alone. In breast cancer, the marker Her 2-neu has been shown to be not only an important prognostic marker, but also a target of therapy [2]. The identification of the protein associated with bcr-abl (break point cluster region-Abelson proto-oncogene fusion) led to a major treatment breakthrough in chronic myelogenous leukemia (CML) [3]. Given these successes, interest has been generated in discovering molecular markers that would help with prostate cancer. Numerous markers have been evaluated, but usually in small numbers and in diverse patient populations. Also, they rarely are evaluated as to whether they enhance the predictive ability of the standard markers. The real test of a new maker (and the key to its success) will be whether it enhances the predictive ability of the prognostic triad of PSA, Gleason score, and stage (with all of its various subclassifications). The purpose of this evaluation is to determine whether any of these are truly helpful in determining who will fail after radical prostatectomy and whether we should consider adding them to our standard armatorium for evaluation.

## 2. Materials and Methods

 A comprehensive Medline search was undertaken to identify studies of molecular markers in prostate cancer. In each of those studies, references were evaluated to try to capture all the studies that evaluated markers in patients undergoing radical prostatectomy.

 Most studies had too few patients to have enough statistical power to make meaningful prognostic statements. Also, most studies were not specific for patients that underwent surgery for their prostate cancer, but rather a mixture of different treatment modalities. While we reviewed all the studies, we focused on those with radical prostatectomy patients that were treated with curative intent. We also focused on studies with more than 50 patients, with the premise that lesser numbers were unlikely to have statistical relevance. In addition, we hoped to focus on studies that evaluated the investigated markers in conjunction with at least one of the accepted predictive factors (PSA, Gleason score, and stage). As it turned out, direct correlation with the known predictive factors was not very commonly performed. 

 We searched for studies that show the possibility of increasing the predictive ability and discuss whether any appear to be able to help us better predict failure. We were not so much interested in determining the mechanistic underpinnings of cancer development and the effect on stage, rather whether markers could help predict the clinical behavior of prostate cancer and help determine appropriate intervention to try to cure more patients.

## 3. Results and Discussion

The literature was quite diverse, which makes interpretation of results difficult and direct comparisons impossible. As per all retrospective studies, there is inherent variation in the selection of patients. In addition, even for the same markers, the determination of positive and negative often varied greatly. Many of them were determined by semiquantitative immunohistochemical (IHC) staining with large methodological and intraobserver variability. Some investigators acknowledged that the staining level to determine what was “positive” had to be manipulated to have significant results [4]. Many studies included patients that received neoadjuvant androgen ablation or adjuvant androgen ablation and/or radiation therapy. While excluding those patients eliminates some of the perceived higher-risk patients, including patients that have additional treatments known to alter failure is also problematic. Also, most of the studies have very short followup, which makes any conclusions about failure tenuous at best. Many expressed markers show a close association with known prognostic factors and while they may be positive on univariate analysis, they fall out on multivariate analysis. These issues are inherent to retrospective studies, but they should be kept in mind.

### 3.1. Ki-67

Ki-67 is one of the earliest markers and is named for the original mouse antibody researched in Kiel Germany, reacting in well number 67 [5]. It serves as a proliferation marker that occurs only in dividing cells (not G_0_). The original antibody required fresh tissue, but the MIB-1 antibody can be used in formalin fixed tissue. The assessment of Ki-67 gives an estimate (index) of the portion of cells actively proliferating. 

Some studies report that Ki-67 is prognostic for failure (Table 1). In a study of 70 radical prostatectomy patients, 50 were selected for further analysis [6]. With a median followup of 63 months, 18% failed (PSA > 0.2 ng/mL). The specimens were evaluated for Ki-67 via IHC staining. On univariate analysis of PSA, PSA doubling time, Ki-67%, tumor volume, and Gleason score, only Ki-67% and PSA were significant prognostic factors. In another study [7], 137 patients underwent radical prostatectomy with a mean followup of 5.4 years. The cohort included 25% lymph node positive and 36% received adjuvant therapy with radiation and/or androgen ablation. Ki-67 was scored as the per cent of staining >5% (78 or 57% if the patients) or <5% (59 or 43% of the patients); the mean was 7.5%. From the graph, for patients below the mean staining, 5-year recurrence free survival was approximately 78% compared to 65% if above the mean. Ki-67 was a significant factor on multiparameter analysis. The largest study evaluating Ki-67 was of 528 prostatectomy patients after exclusion of those that received neoadjuvant and adjuvant androgen ablation and radiation therapy [8]. With a median followup of 46 months, 101 (19%) failed for a 5-year disease-free (PSA < 0.2 ng/mL) rate of 78%. The tissue was evaluated using IHC staining for Ki-67 and chromogranin A (CGA). On multivariate analysis, Gleason score > 4 + 3, CGA positive, lymph node positive, PSA > 20 ng/mL, and Ki-67 were prognostic, while pathologic stage T3 and margin positivity were not. For the 300 Ki-67 ≥ 5% patients, 5-year biochemical recurrence-free survival (from graph) was 70%, while for the 228 with <5% staining, it was 88%. In another large study, Miyake et al. [9] studied 193 prostatectomy patients that did not receive adjuvant treatment. With a median followup of 63 months, 21% failed for a 5-year disease-free survival rate of 79%. They evaluated twelve markers with IHC. On univariate analysis, they found the following factors to be prognostic: PSA, Gleason score, lymph node positivity, tumor volume, seminal vesicle involvement, margin positive, and on immunohistochemical staining: Ki-67, p53, AR, MMP-2, MMP-9, and HSP27. On multivariate analysis, only Ki-67, seminal vesicle involvement, and margin positivity remained significant. In consideration of those three positive factors, if the patient was positive for 2 or 3 of them, the recurrence rate was 79%, if positive for one, 20%, and if negative for all 3, 4%. Ki-67 was also prognostic in a smaller study of 91 prostatectomy only patients [10]. With a median followup of 46.5 months, 29 (32%) progressed (PSA ≥ 0.2 ng/mL). For the 60% of patients with <5% PSA staining, 5-year disease-free survival was 84%, compared to 42% for those with ≥5% staining (from graph). On multivariate analysis, Ki-67 and Gleason score were prognostic. The final positive study was a multifactorial study [11] of 336 RRP patients, of which 249 had tissue. Lymph node positive patients were included. Failure was defined as PSA > 0.5 ng/mL. Five-year DFS was 63%, and 10-year was 41% with a median followup of 66 months. They utilized immunohistochemical staining for Ki-67, enhancer of zeste homolog 2 (EZH2), (discussed below) and minichromosome maintenance protein 7 (MMC7) (discussed below). They also used fluorescence in situ hybridization (FISH) for EIF3S3, a chromosome abnormality they had explored previously. On multivariate analysis considering EZH2, Ki-67, MCM7, Gleason score, pathologic stage, and PSA, the factors of pathologic stage, Ki-67 and MCM7 were significant predictive factors. From the graphs, for staining 0-1%, 10-year disease-free survival was 65%, for 2–15% was 38%, and for >15% was 27%. Demonstrative as to how other factors can have an effect of prognostic ability, in patients that were lymph node negative with an undetectable postsurgery PSA, Ki-67 dropped out and EZH2, MCM7, and PSA were prognostic. In Gleason, less than 7 patients, Ki-67 was the only significant factor; the 15 patients with Ki-67 staining of >1% had a 5- and 10-year disease-free survival of 70% and 45%, respectively (from the graph), compared to 100% for Ki-67 of 0-1%. No details of interaction with pathologic variables were given.

Even though those studies showed on multivariate analysis Ki-67 was able to predict failure, other than the correlation shown in the Miyake et al. study [9], none of the studies evaluated as to how Ki-67 improved the predictive ability of the standard prognostic factors. Therefore, its utility remains uncertain, which is further compounded by the studies that show that Ki-67 is not predictive for failure. In that regard, in a study of 162 patients undergoing RRP (median followup 4.5 years, PSA failure > 0.2 ng/mL at least twice), Ki-67 staining was measured <2 in 62% of the tumors and 2–4 in 38% [4]. On multivariate analysis including pathology stage, race, Gleason score, age, p53, bcl2, and Ki-67 (MIB-1) levels, p53 and bcl-2 were prognostic, but not Ki-67. The findings were confirmed in another study of the same patients [12]. From the cohort of 335 patients, this time 180 had available tissue. With a mean followup 4.4 years, and failure defined as PSA > 0.2 ng/mL twice, the overall 5-yr biochemical failure-free survival (BFFS) was 60%. Ninety per cent had measurable Ki-67 (MIB-1) staining. In 18 patients with negative or rare Ki-67 staining, 3 (5%) progressed for an 83% 5-year biochemical-free survival (BFFS); of 90 that stained 1+, 23 (37%) progressed for a 69% 5-year BFFS; and in 72 that were 2–4+, 36 (58%) progressed with a 5-yr BFFS of 44%. On multivariate analysis, stage and Gleason score were significant prognostic factors and Ki-67 was only marginal. In a subgroup analysis, Ki-67 appeared to differentiate failure in Gleason 2–6 patients, but not in higher grade. A third paper including at least some of the same patients (132) [13] showed Ki-67 positive patients had a higher recurrence rate but again the findings were not significant on multivariate analysis. In a different approach [14], 41 prostatectomy patients who failed within two years (PSA > 0.2 ng/mL) were matched for pathologic stage, PSA, and Gleason score with 41 patients who did not have a rising PSA by three years. They found no difference in Ki-67, p53, and bcl-2 between the two groups. Finally, in an evaluation of 112 prostatectomy patients [15], for patients with low MIB-1 staining, the 5- and 10-year clinical disease-free survival was 75% for both, and for high staining patients was 52 and 42%, respectively. In spite of this difference, MIB-1 was not predictive of recurrence or death on multivariate analysis.

### 3.2. Apoptosis-Related Markers (p53, bcl-2, and MDM2)

Cellular stress triggers (upregulates) p53, which accumulates in cells and leads to either cell cycle pause and repair or apoptosis. Loss of p53 function potentially can allow a cell that would normally undergo apoptosis to survive an otherwise lethal event. Bcl-2 is antiapoptotic and elevated levels can also conceptually allow cells to survive an otherwise lethal event. Mouse double minute-2 (MDM2) has an antiapoptotic effect by binding to p53 and inactivating it. Wild-type or normal p53 is cleared rapidly from cells, so measurable p53 is usually dysfunctional. Therefore, counter-intuitively, an elevated p53 actually represents decreased p53 function. 

 As with Ki-67, there are several positive and negative studies (Table 2). In 71 patients operated on before 1984 [16] with a median followup of 10.6 years, 15-year cause-specific survival for p53 positive patients was 38% and for p53 negative patients was 87%. They also found that the 15-year cause-specific survival for retinoblastoma protein (Rb) positive patients was 66% and Rb negative was 92%. On multivariate analysis, the combination of p53 and Rb was the strongest predictor of failure. There was no analysis with the common prognostic factors (stage, PSA, or Gleason score). A later study in 76 RRP patients with a median followup of 50 months found that 27% of the patients with <40% positive p53 staining recurred versus 6/10 (60%) with more than 40% staining [17]. On univariate analysis, nuclear grade, pathologic stage, and p53 were significant, but on multivariate analysis, only p53 was significant. In another prostatectomy study, 263 patients had a mean followup of 55 months and 39% failed [18]. Seventy-eight received adjuvant treatment. They found clustering of p53 positive cells (>12 cells) to be more predictive than percentage of positive cells. On multivariate analysis, both clustering and percentage p53 positive, along with PSA, path stage, Gleason score, and lymph node positivity were predictive for failure. 

 Several studies have considered p53 in conjunction with other factors such as bcl-2, and Ki-67. In one study consisting of 162 patients undergoing RRP (median followup 4.5 years, PSA failure > 0.2 ng/mL at least twice) p53 was measured negative in 31% of the tumors and positive (1–4+) in 69%. Bcl-2 was measured negative in 73% of the tumors and positive (1–4+) in 27% [4]. On multivariate analysis including pathology stage, race, Gleason score, age, p53, bcl-2, and Ki-67 (MIB-1) levels, p53 and bcl-2 were prognostic. There was no correlation as to what the markers added to the common prognostic markers. In another study from the same patient cohort, 175 patients underwent radical prostatectomy [19]. With a mean followup of 4.6 years, p53 staining was positive in 65% and the 5-year failure rate was 51%, compared to 22% for the patients that stained negative. Bcl-2 staining was positive in 27% and the 5-year failure rate was 67%, compared to 31% for the patients that stained negative. For patients that were both p53 and bcl-2 positive, the five-year failure rate was 75% compared to 20% for those that were negative for both. On multivariate analysis, stage, race, bcl-2, and p53 were all prognostic. Again, there was no indication of whether they enhanced the standard markers. Interestingly, in yet another analysis of some of the same patient cohort (132 patients) with median followup of 3.9 years, p53 positive patients had a higher recurrence rate but it was not significant on multivariate analysis [12]. Another study of p53 and bcl-2 looked at 76 prostatectomy patients with a mean followup of 38 months, 23 (30%) of whom failed [20]. Fifty-seven percent were p53 positive on prostatectomy tissue and 41% failed compared to 21% with normal p53. Twenty percent were bcl-2 aberrant on prostatectomy tissue and 53% failed compared to 24% of those with normal bcl-2. In an additional study of 119 radical prostatectomy patients receiving no neoadjuvant treatment and with a median followup of 3.3 years, 16 (13%) failed [21]. On multivariate analysis, bcl-2, p53, Ki-67, PSA, Gleason score, Capsular penetration, age, and margin positivity were not predictive, but SV involvement and caveolin-1 (see below) were. 

 In an older cohort of patients (22% of the failures predated PSA), 30 received adjuvant treatment (mostly radiation) [22]. With a mean followup of 5.2 years, bcl-2 positivity was predictive of recurrence, but only stage pT3 and Ki-67 were predictive of failure (not p53). From the graph, for elevated bcl-2, 10-year disease-free survival was 18% and for nonelevated bcl-2 was 52%. Only 8% overexpressed p53. Like p53, it is also not uncommon for bcl-2 staining to be too low (<5%) to be meaningful [10]. 

 Miyake et al. evaluated 193 prostatectomy patients with twelve markers on IHC, including p53 [9]. While it was predictive on univariate analysis, it was not on multivariate. Bcl-2 was not predictive for either. In a study of 70 pathological T2 patients [23] with a median followup of 36.5 months, 30% suffered biochemical relapse (PSA > 0.2 ng/mL times two), sixteen patients were p53 positive, and 44% suffered PSA relapse which was not significantly different than the p53 negative patients (26% relapse). Only 3 (4%) patients were bcl-2 positive, but 2 (67%) relapsed, which was significantly higher than the bcl-2 negative patients (28% failure). Finally, in a study [24] of 86 patients (median followup 65 months) with an undetectable PSA after radical prostatectomy (38% received neoadjuvant treatment), 20% overexpressed p53 and had a higher risk of relapse. The 33% that overexpressed MDM2 also had a higher risk of relapse. No details were given, but on multivariate analysis, both p53 and p21 were predictive. Stage and MDM2 were not. Interestingly, there was no association with p53 overexpression and p21 or MDM2. As with all the studies discussed, there was no real analysis for correlation with standard predictive factors, so the real predictive power of these markers remains elusive.

### 3.3. E-Cadherin and Other Adhesion Molecules

Calcium-dependent adhesion molecules (cadherins) are transmembrane proteins that play a role in cell adhesion. E-cadherin is a subtype found in epithelial tissue with extracellular, transmembrane, and intracellular domains. The intracellular domain binds to beta catenin. In cancer, E-cadherin downregulation theoretically reduces cell adhesion resulting in increased cell motility and dissemination. 

 In a study of 70 pathological T2 patients [23] with a median followup of 36.5 months, 30% suffered biochemical relapse (PSA > 0.2 ng/mL times two). Thirty-nine patients (56%) had aberrant E-cadherin staining, with a 44% PSA relapse rate, which was significantly worse than those with normal E-cadherin staining (13% recurrence). In 104 prostatectomy patients [25] (7 lymph node positive), low E-cadherin, Gleason score, and pathologic stage were predictive of biochemical failure (PSA > 0.5 ng/mL) on multivariate analysis. For clinical failure, pathological stage dropped out and elevated N-cadherin was significant. For patients with low E-cadherin, the 10-year biochemical failure-free survival was 14%, versus 33% for those with elevated levels. For N-cadherin, low levels resulted in 33% biochemical failure-free survival and high levels 14%. They found that the E-cadherin to N-cadherin ratio was more powerful than either alone, but did not provide specifics nor any details on the modification of the predictive power of standard factors. In a study of 67 radical prostatectomy patients [26] with a median followup of 54 months, 27 (40%) recurred clinically, 7 locally, and 20 systemically. When evaluated with IHC for E-cadherin, a cut point of 40% staining was chosen. For the 13 that stained less than 40%, 2 (15%) died of cancer and for the 54 that stained >40%, 14 (26%) died of cancer, but the difference was nonsignificant. E-cadherin was not predictive on univariate or multivariate analysis for either recurrence or survival. In 128 radical prostatectomy patients [27] without adjuvant treatment, tissue microarrays were made and stained with IHC staining. Normal was considered >70% staining. For nonmetastatic prostate cancer, 18% had aberrant staining. With a median followup of 23 months, 38% of the failures and 20% of the nonfailures had aberrant staining, a nonsignificant difference. Similarly, in a microarray study (discussed below), Rhodes et al. [28] found that a decreased E-cadherin to EZH2 ratio resulted in an increased rate of biochemical failure after radical prostatectomy. 

 Brewster et al. [20] studied 76 prostatectomy patients; 49% were E-cadherin aberrant on prostatectomy tissue and 37% failed compared to 22% with normal E-cadherin. On multivariate analysis, it was not predictive when considered with p53, bcl-2, Gleason score, and margins. They also evaluated another apparent adhesion molecule in the form of the cell surface glycoprotein CD44. Sixty-four percent were CD44 minimal or absent on prostatectomy tissue. Of those with normal staining, 8% failed compared to 43% with aberrant staining. On multivariate analysis, it was not predictive when considered with p53, bcl-2, Gleason score, and margins. Two other studies evaluated CD44. In 97 radical prostatectomy patients [29] with median followup of 84 months, utilizing PSA of >1.0 as failure, most (86%) patients were positive for CD44, so risk was determined by graded intensity of the staining. Decreased expression increased the risk of failure. On univariate analysis, loss of CD44 and cd4v6 were predictive of clinical failure, but only CD44 was predictive for biochemical failure. In the other study, 99 patients had mean followup of 40 months and 26% suffered a biochemical recurrence [30]. CD44 was evaluated via an intensity and percent staining score, and 47% were downregulated. The 3-year recurrence-free survival was 77% for the nondown-regulated patients versus 48% for those with CD44 downregulation. It was not a significant predictor on multivariate analysis, when considered with p34. 

 In none of these studies was there an assessment of how it modified the predictive ability of the standard prognostic factors.

### 3.4. EZH2

The Enhancer of Zeste 2 (EZH2) gene codes for polycomb group proteins that effect chromatin and silence genes. When overexpressed, it appears to be associated with tumorigenesis. In a study involving multiple cancers [31], 104 radical prostatectomy patients with a median followup of 104 months were evaluated with staining for EZH2. For low EZH2 staining, the 5- and 10-year cause-specific survival was 99% and 93%, respectively. For the high staining group, it was 89% and 53%, respectively. On univariate analysis, upper quartile EZH2 staining was predictive for clinical recurrence and on multivariate analysis was predictive for distant metastasis and death. In another study of 64 patients [32], tissue was stained for EZH2 and if the intensity was ≥3, 10/32 (31%) failed versus 3/32 (9%) if the staining was <3. It was a significant factor on multivariate analysis along with margin status, tumor size, Gleason score, and PSA. Finally, in a study (see Ki-67 above) [11] of 249 prostatectomy patients, five- and 10-year disease-free survival was 63% and 41%, respectively. On multivariate analysis, pathologic stage, Ki-67 and MCM7 were significant predictive factors (EZH2 was not). In patients that were lymph node negative with an undetectable postsurgery EZH2, MCM7 and PSA were prognostic. In Gleason less than 7 patients, Ki-67 was the only significant factor. There was no evaluation of whether this added to the predictive ability of standard factors.

### 3.5. Cyclin-Dependent Kinases (and Their Effectors)

Cyclin dependent kinases (CDKs) are protein kinases involved in the regulation of the cell's progression though the cell cycle. As most cancers have dysfunctional cell cycle control, the kinases are implicated as part of the aberrancy. Cyclin D1 is specific for transition through G1/S. It has its effect by binding with cyclin dependent kinases 4 and 6 forming a complex that phosphorylates and inactivates the retinoblastoma protein (Rb). Overexpression of cyclin D1 has been associated with the malignant phenotype and its progression. There are several known inhibitors of cyclin dependent kinases. For example, p16INK4a (cyclin-dependent kinase inhibitor 2A) inactivates Cdk4 and CdK6 and thereby acts as a tumor suppressor (by blocking the phosphorylation of the Rb gene, which prevents transit through G1). Loss of p16 enables abnormal progression through the cell cycle, increasing the malignant potential. P21-waf1 encodes a cyclin dependent kinase inhibitor (p21 or cyclin dependent kinase inhibitor 1A), inhibiting CDKs 2 and 4, which leads to arrest at G1. It is induced by p53 (thus elevated p53 can lead to arrest at G1 through this route). P27Kip1 (cyclin dependent kinase inhibitor 1B) is also involved in G1 arrest by inhibiting cyclin dependent Cdk2 complexes E and A and D-Cdk4. Therefore, a decrease in p27 should result in increased proliferation. Lastly, p34cdc2 (cell division control protein 2) is a component that forms a kinase by binding with cyclin B1 (forming maturation-promoting factor (MPF)) that regulates G2/M transition and promotes mitosis).

 In a study [24] of 86 patients with an undetectable PSA after radical prostatectomy (38% received neoadjuvant treatment), 33% overexpressed p21Cip, and this was associated with a higher risk of relapse. No details were given, but on multivariate analysis, both p53 and p21 were predictive of relapse whereas stage and MDM2 were not.

 In one study, where the primary goal was to assess the association between pathological features and biomarker expression [33], p27Kip expression was evaluated in 113 prostatectomy specimens (median followup 4.6 years, 21% neoadjuvant androgen ablation), and correlated with outcome. Low p27 nuclear staining was a poor prognostic sign. On multivariate analysis, p27, seminal vesicle status and margin status were all predictive for recurrence, but no details were given. In a second study of 96 stage C lymph node negative patients undergoing radical prostatectomy with a median followup of 9.5 years [34], p27 Kip1 staining correlated with Gleason score (higher grades had decreased levels). The 9-year recurrence-free survival for levels ≤10% was 17%, for levels 11–50% was 47%, and for >50% was 67%. There was no correlation with the standard factors. In a third p27 study [35] of 86 patients (after excluding those that received adjuvant treatment), multivariate analysis demonstrated only pathologic stage and p27 to be predictive at a median followup of 40 months. High Gleason score was associated with low p27 staining. Thirty percent was the breakpoint between high and low staining. Fifty percent of patients with low staining failed (PSA > 0.4) and 78% with high staining failed. In another study with 95 patients [36], loss of p27 (<10%) on multivariate analysis was significant for recurrence, but not for survival. With a median followup of 49 months, 33% of the p27 negative patients failed versus 23% for the p27 positive patients (median followup 59 months). Another study was of 161 prostatectomy patients [37], which were divided into organ confined (*n* = 76, median followup 42 months) and nonorgan confined (*n* = 85, median followup 38 months) patients. p27 staining was performed on the biopsy, but not the final pathology specimen, and patients were not evaluated for the specific impact of positive margins, seminal vesicle involvement, or lymph node involvement. For the organ-confined patients, the 5-year recurrence rate was 26%, but 9% for those with high p27 staining and 37% with low (<45%) staining. In this subgroup, p27 was predictive for failure. In the nonorgan confined patients, the recurrence rate was 44%, but p27 was not predictive of failure in these more advanced patients and the actual effect on failure was not stated. In an evaluation [15] of 112 prostatectomy patients, 92 had adequate p27 staining. Thirty-five (38%) stained less than 50% and were classified as low staining. Based on clinical parameters, their 5- and 10-year disease-free survival were 37% and 26%, respectively. For the high staining patients, it was 79% and 77%, respectively. p27 predicted for clinical recurrence and cause-specific survival.

Finally, in a study of 104 radical prostatectomy patients with a median followup of 56 months [38], p27 was determined by the per cent of nuclei staining, with the median of 64% used as the breakpoint between high and low. On multivariate analysis, pathologic stage and PSA were significant predictors of recurrence, but not p27. 

 p16 has been evaluated in several studies. In 206 radical prostatectomy patients (18% with neoadjuvant androgen ablation) with a median followup of 72 months, one group [39] found positive p16INK4a staining to be associated with recurrence. On multivariate analysis, p16, PSA, Gleason score, and margin status were all predictive, but no actual outcome data was given. In another study [40], 88 prostatectomy patients (39% neoadjuvant treatment) with a median followup of 65 months stained for P16. Unlike Henshall et al. [39] (which called low <1%), their breakpoint was 5% positive nuclear staining. For the 38 patients that overexpressed, 21 (55%) failed versus 26% of the 50 under expressing patients. p16 was associated with PSA levels and was not an independent prognostic factor on multivariate analysis. They also did not report specifics on outcome. In a third study of 104 radical prostatectomy patients with a median followup of 56 months [41] the multivariate analysis for survival was positive for p16, age, grade, capsular penetration, and seminal vesicle involvement. They scored p16 by a fluorescence index. The low group had a 5-year survival of 78% versus 43% for the intermediate group (*P* = .005) and 42% for the high index group. There was no outcome data accounting for the standard factors. Ploidy or S phase was not predictive. 

In analysis of cyclin D1 and p34cdc2, 140 patients [42] with a median followup of 42 months were evaluated. Failure was defined as a PSA > 0.4. In patients that were p34cdc2 negative, 10% failed versus 26% that were positive. For Gleason 7 or greater, the failure rate was 26% for p34cdc2 negative and 38% for positive. On multivariate analysis, only p34cdc2 and Gleason score were predictive and cyclin D1 and ploidy were not. p34 was also evaluated in a study of 99 patients. With a mean followup of 40 months, 26% suffered a biochemical recurrence [30]. p34 was evaluated via an intensity and percent staining score and 61% were determined to have overexpressed p34. The 4-year recurrence-free survival (from the curves) was 98% for the nonover expressed patients versus 47% for those over expressing p34. It was a significant predictor on multivariate analysis, but there was no evaluation of whether it enhanced the predictive ability of standard factors.

### 3.6. Cathepsin-D

Cathepsins are proteases (i.e., involved in protein degradation) usually housed in lysosomes that proteolyse proteins that regulate cell growth. In a study [43], 105 radical prostatectomy patients were evaluated for cathepsin D. It was not prognostic on either univariate or multivariate analysis, but probably because the expression rate was extremely high at 98%.

### 3.7. Chondroitin Sulfate

Chondroitin sulfate is a structural glycosaminoglycan of the extracellular matrix that helps regulate cell activity. Ricciardelli et al. [44] studied 157 prostatectomy patients after exclusion of adjuvant and neoadjuvant treatment; failure was defined as a PSA > 0.2 and median followup was 47 months. They used an antibody to chondroitin sulfate and read the slides via an image capture technique with automated analysis. There was a twofold difference between this study and previous studies for the absolute value of the mean due to calibration differences, which demonstrates the lack of uniformity in these studies. The median was chosen as the cut point, although the most robust point was slightly above that. On multivariate analysis, chondroitin sulfate, Gleason score, preoperative PSA, and pathological stage were all predictive. For patients with low staining, 23% failed for a 5-year PSA failure rate of 33% versus 51% with high staining failing for a 5-year failure rate of 51%. There was some correlative analysis between chondroitin staining and other predictive factors. For patients with a preoperative PSA less than 10, 9% with low chondroitin sulfate staining failed versus 48% with high levels. In a more specific analysis, the five-year failure rate for Gleason 5–7 patients with low chondroitin levels and low PSA was 11% compared to 44% for low chondroitin staining patients with a high PSA. Further, Gleason 5–7 patients with high chondroitin sulfate staining and low PSA had a five-year failure rate of 56% versus 72% for high staining and high PSA. There was no evaluation done with the integration of pathology findings.

### 3.8. Hepsin and PIM1

Hepsin is a transmembrane serine protease whose exact function is unknown, but when upregulated appears to express a malignant phenotype. PIM1 encodes a protein kinase that promotes G1/S transition by upregulation of CDK2, facilitating cell proliferation and survival. One study [45] utilized human specimens and cell lines for comparison of malignant and benign tissue. Out of several hundred candidate genes, hepsin and PIM1 expression proteins were selected for further analysis. Hepsin was increased in malignant prostate tissue versus benign, but staining was greatest in PIN. In radical prostatectomy patients, low or absent hepsin increased failure. On multivariate analysis, both hepsin and Gleason score were predictive of failure. They also tested for PIM1. It was upregulated in prostate cancer and decreased levels were associated with increased PSA level in 135 patients with localized prostate cancer. It was significant on multivariate along with Gleason score 4-5 and PSA. They concluded that lower PIM1 levels were strongly associated with an increased risk of relapse. There was no outcome correlation with standard factors with either marker.

### 3.9. Cox-2

In a study of 91 prostatectomy patients [10], with a median followup of 46.5 months, 29 (32%) progressed (PSA > 0.2 ng/mL). A score was developed for percent and intensity of staining for Cox-2. For no staining, the failure rate was 26% versus 60% for 1–4, but then dropped back to 15% for 5–12. While it was a predictive marker on univariate analysis, it was not on multivariate.

### 3.10. Laminin Receptor (Ribosomal Protein SA)

Laminins are glycoproteins located in the basement membrane (basal lamina) that affect cell adhesion and migration as well as differentiation and survival. Laminin receptor (LR) is detected via the MLuC5 antibody. In an initial evaluation [46] in 140 patients, it appears that laminin receptor positivity might be associated with recurrence. Overall, the 3-year biochemical failure-free survival was 68%, but for LR positive patients the failure was 45% and for negative patients it was 7%. There was no correlation with PSA and Gleason score. The followup was only 20 months, and a later paper [47] showed that LR measurement of the biopsy tissue was not significantly predictive for biochemical progression, probably due to a lack of concordance between the measurements in biopsy tissue versus the larger tumor specimen.

### 3.11. Chromogranin A (CGA)

In a study of 528 prostatectomy patients [8] excluding neoadjuvant and adjuvant androgen ablation and radiation therapy, with a median followup of 46 months, 101 (19%) failed for a 5-year disease-free (PSA < 0.2 ng/mL) rate of 78%. The tissue was evaluated using IHC staining for Ki-67 and chromogranin A (CGA). On multivariate analysis, Gleason score > 4 + 3, CGA positive, lymph node positive, PSA >20 ng/mL, and Ki-67 were prognostic, while pathologic stage T3 and margin positivity were not. For the 32 CGA positive patients, the 5-year biochemical recurrence-free survival was 48% and for the 496 CGA negative it was 80%. Because of the small number of CGA positive patients, the only specific information was given on whether there was modification of prognosis of the standard factors was for Gleason <7 patients, where for the 304 CGA negative patients, 8% failed and for the 12 CGA positive, 25%.

### 3.12. Minichromosomal Maintenance Protein 7 (MCM7)

Minichromosome maintenance protein 7 (MCM7) appears to be a facilitator of DNA replication, so upregulation would be expected to increase proliferation. It has been found on microarray analyses that MCM7 is frequently amplified in prostate cancer. In an evaluation of prostatectomy patients [48], 52/68 (77%) with MCM7 amplification relapsed versus 7/57 (12%) without amplification. In a study discussed above (see Ki-67) [11], pathologic stage, Ki-67, and MCM7 were significant predictive factors. In evaluation of patients that were lymph node negative with an undetectable postsurgery, EZH2, MCM7, and PSA were prognostic. In both studies, there was no clinical correlation, so these interesting findings are of uncertain significance.

### 3.13. Histones

Histones are intranuclear proteins in chromatin around which DNA is “wound”, the modification of which influences their interaction with the DNA and affects some processes such as mitosis and gene regulation. In 183 radical prostatectomy patients, those that received androgen ablation were excluded. The median followup was 60 months and failure was defined as PSA > 0.2 ng/mL [49]. In order to evaluate sites on histones H3 and H4 with acetylation and dimethylation staining, 5 different sites were identified by using a clustering algorithm. While not independently predictive, when combined with Gleason score, the findings yielded prognostic information. From the graph, Gleason < 7 patients that were histone “favorable” had an 84% disease-free survival, while those unfavorable had a 58% disease-free survival. For Gleason 7–10, the favorable group had a disease-free survival of 46% versus 20% for the unfavorable.

### 3.14. TMPRSS2 : ERG Fusion

TMPRSS2 (transmembrane protease, serine 2) is an androgen-regulated gene found on chromosome 21 that encodes a transmembrane protease. In prostate cancer, it can be fused with genes for the ETS transcription factors, such as ERG (resulting in TMPRSS2 : ERG). This indirectly places ERG under androgen transcriptional control. There are multiple variants of this fusion. This can be detected through either RT-PCR or fluorescence in situ hybridization (FISH). In 165 prostatectomy patients with available frozen tissue [50] with a median followup of 20 months, tissue was evaluated for TMPRSS2 : ERG fusion gene and 49% was positive. For the fusion gene positive patients, 46% failed compared to 7% in fusion negative patients. On multivariate analysis, the fusion gene was the most predictive factor, followed by grade. Evaluation was made for different Gleason and pathologic findings. For Gleason 5-6 patients, 33% of the gene positive patients failed, versus 5% for the gene negative. For Gleason 7 and Gleason 8–10, it was 48% versus 7% and 75% versus 14%, respectively. For organ-confined patients, gene positive patients had a recurrence rate of 34% versus 10% for gene negative. For extraprostatic extension and seminal vesicle positive patients, it was 53% versus 3% and 67% versus 34%, respectively. For both Gleason score and pathological findings, all the differences were statistically significant, except for the seminal vesicle involved patients. Another study, started with 248 radical prostatectomy patients [51], but only 150 were ultimately evaluable by FISH. Of those, 50 (33%) were found to have TMPRSS2 : ERG rearrangement. With a median followup of 66 months and failure defined as two rises of PSA > 0.5 ng/mL, on multivariate analysis, Ki-67, pathologic stage, and TMPRSS2 : ERG fusion were significant, not Gleason score or PSA [52]. Yoshimoto et al. evaluated specimens from 125 radical prostatectomy patients, 122 of which had clinical followup and with a median followup, 49% had failed (PSA > 0.2 ng/mL). Neoadjuvant androgen ablation was allowed, and 2 patients were lymph node positive. FISH was used to evaluate for TMPRSS2 : ERG, and 48% were found to have rearrangements resulting in a 5-year biochemical failure-free survival (BFFS) of 46%. For those that were negative, 5-yr BFFS was 62% (*P* = .0523). Expanding on their previous work, they also evaluated for PTEN deletion by FISH. Only 82 of the 125 patients could be evaluated. There was no difference in 5-yr BFFS between those that were deletion negative and positive, but if they divided the deleted patients into hemizygous and homozygous deletions, they found that all the homozygous patients had failed by 5 years. If patients had both the PTEN deletion and the TMPRSS2 : ERG fusion, 5-yr BFFS was 30% versus 59% if they had neither (*P* = .001). They did not test to see whether these markers augmented the predictive ability of the three standard factors (Stage, Gleason score, or PSA), although on multivariate analysis only Gleason score, the TMPRSS2 : ERG/PTEN combination and homozygous PTEN deletion were prognostically significant. A study [53] using microarray to compare genes between benign and malignant cells found that ERG was the most commonly over expressed. Then utilizing QRT-PCR, they analyzed 114 prostate cancer patients and found ERG1 over expressed in 62%. Ninety-five patients had detectable levels and for a >100 over expression, the 5-year biochemical failure free-survival (from the graph) was 88%, for 2–100 fold 80% and for <2 fold 36%. On multivariate analysis, ERG1 (>100 versus <2) and Gleason (8–10) were significant, but not race, PSA, pathologic stage, margin positive, or seminal vesicle positivity.

 Not all studies found TMPRSS2 : ERG to be prognostic. In one study [54], two subgroups were taken from larger prospective studies and ultimate outcome collected from SEER data. This yielded no failure data and only crude followup of cancer-specific survival. Of the subgroups, only 57% could be scored for the fusion. They reported no association between the occurrence of TMPRSS2 : ERG (positive in 36% of the patients) and cancer specific survival. Researchers in a study [55] of 521 radical prostatectomy patients with 95 month median followup utilized FISH and found 42% had TMPRSS2 : ERG abnormalities. It was not associated with recurrence, metastasis or death. Finally, in a study of 54 patients [56], 35 (65%) had gene rearrangement, which was present in 60% of the nonfailing patients and 65% of the failing patients. In the evaluation of 28 benign prostate tissues, there were no rearrangements.

### 3.15. PTEN

The phosphatase and tensin homologue (PTEN) gene modulates the phosphotidylinositol 3-kinase (PI3K) pathway, a regulator of the Akt pathway. Lack of PTEN allows for upregulation of Akt and other cell cycle factors, increasing cell survival. As noted above [52] in the TMPRSS2 : ERG discussion, on multivariate analysis, homozygous PTEN deletion and the TMPRSS2 : ERG fusion were prognostically significant. In an earlier study specifically evaluating PTEN, the same authors [57] utilized fluorescence in situ hybridization (FISH) to PTEN in 107 prostatectomy patients. Tissue was scored as showing no deletions (56%), hemizygous deletions (39%), or homozygous deletions (5%). On Cox proportion hazard analysis, for univariate analysis, perineural invasion, seminal vesicle positive (SV+), extraprostatic extension (EPE), Gleason score, PSA, lymph node positivity, and PTEN deletion were all predictive. On multivariate analysis, only EPE, SV+, and PTEN were predictive. For PTEN, from the graph, 5-year PSA (>0.2 ng/mL) failure-free survival was 0 for the 5 homozygous patients, 48% for the 42 hemizygous patients, and 60% for the 60 patients without deletion. There was no discussion as to how PTEN modified the predictive ability of standard factors. In a separate study of 104 radical prostatectomy patients with a median followup of 56 months [38], PTEN was scored as an index based on percent staining and intensity. On multivariate analysis, pathologic stage and PSA were significant predictors of recurrence, but not PTEN.

### 3.16. Epidermal Growth Factor Receptors (EGFR)

Epidermal growth factors are extracellular ligands controlled by the cell surface epidermal growth factor receptors, which are tyrosine kinase receptors. When activated, they initiate a cascade of signal transduction (i.e., though the Akt pathway) that results in cell proliferation. If the receptor is mutated in the “on” position (i.e., over expression), the result could be uncontrolled proliferation. Her-2/neu (c-erb B2) encodes a tyrosine kinase growth factor receptor similar to the epidermal growth factor receptors and has been linked with advanced disease. In one study, [43] 105 radical prostatectomy patients were evaluated for epidermal growth factor receptor (EGFR). The expression rate was 48%, but it was not prognostic on either univariate or multivariate analysis. In 113 prostatectomy patients with a mean followup of 42 months [58], utilizing IHC, membranous and cytoplasmic staining was given a composite score so that ≥3 was considered positive. With that parameter, 29% of the tissue over expressed and there was no correlation with failure on univariate analysis. Utilizing FISH, it was found that 41% were amplified for Her2, but there was poor correlation with IHC staining (*P* = .25). While FISH analysis was significant for failure on univariate analysis, it was not a significant predictor of failure on multivariate analysis. In 99 patients with a mean followup of 40 months, 26% suffered a biochemical recurrence [30]. Her 2-neu was evaluated via FISH and 42% were found to be amplified. The 5-year recurrence-free survival was 75% for the nonamplified patients versus 47% for those with Her 2-neu amplification. It was not a significant predictor on multivariate analysis, when considered with p34.

### 3.17. VEGF

In a study of 193 prostatectomy patients [9], twelve markers were evaluated on IHC, including VEGF, but it was not predictive on univariate analysis.

### 3.18. Caveolins

Caveolins are cell membrane proteins involved in endocytosis resulting in invagination of the plasma membrane (caveolae). They appear to be involved in signal transduction with a role in homeostasis and tumorigenesis. Caveolins have been found to be both increased and decreased in cancer so their role is variable and uncertain. In radical prostatectomy patients selected for failing or not failing, 162 lymph node negative patients were identified. With immunohistochemical staining for caveolin 1, 22% were positive and five-year progression-free survival was 43% versus 68% for those that were negative. On multivariate analysis, caveolin 1, Gleason score, extracapsular extension, seminal vesicle involvement, and margin involvement were all predictive [59]. The same group later studied serum levels of caveolin 1. As noted above, in a study [21] of 119 radical prostatectomy patients on multivariate analysis only caveolin 1 staining and SV involvement were predictive on multivariate analysis, but bcl-2, p53, Ki-67, PSA, Gleason score, Capsular penetration, age, and margin positivity were not. For caveolin 1 positive patients, 9/32 (28%) failed versus 7/87 (8%) that were negative. In 232 prostatectomy patients that included lymph node positive and those that received salvage radiation therapy [60], with a median followup of 70 months, the 5-year biochemical-free survival rate was 80%. On multivariate analysis, only Gleason sum (not Caveolin 1 staining) was a significant predictor of failure. When limited to lower risk patients (*n* = 177) with exclusion of lymph node positive, seminal vesicle positive, Gleason > 7, and extracapsular extension/margin positive, caveolin 1 was still not a significant predictor on multivariate analysis. They did find that in evaluating only the recurring patients, those that had caveolin 1 over expression did worse. In a similar study [61], 30% of 152 radical prostatectomy patients (including lymph node positive) stained positive for caveolin 1. It was not predictive on multivariate analysis (only seminal vesicle positivity, margin positivity, and PSA were), but when restricted to patients with organ-confined disease, it was the lone predictive factor. This is somewhat in contradistinction to the low risk patients noted in the study above.

### 3.19. Zinc-Alpha2-Glycoprotein (AZGP1)

Zinc-alpha2-glycoprotein (AZGP1) encodes for a protein historically thought to be involved in lipolysis and thought to have a role in the cachexia of cancer. From a series of 732 radical prostatectomy patients [62], 228 were analyzed. Forty-three percent failed with a PSA rise of ≥0.2 ng/mL. On IHC, tissue was scored as absent or weak versus strong AZGP1 staining. Twenty-nine percent stained weak. Although there were few events, it appears to be predictive of clinical recurrence and distant metastasis, but there was no evaluation as to modification of common prognostic factors. In a gene array study [63] discussed below, AZGP1 was predictive for nonrecurrence.

### 3.20. Alpha Methylacyl CoA Racemase (AMACR)

Alpha methylacyl CoA racemase (AMACR) is a catalytic enzyme (of fatty acids) that is frequently over expressed in prostate cancer, but levels are decreased in advanced cancers as compared to localized. In 204 radical prostatectomy patients [64], IHC was performed for AMACR expression proteins and regression analysis was used to correlate staining with PSA failure (>0.2 ng/mL). With visual scoring on a scale of 1–4, there was no correlation with failure, but with quantitative expression analysis, patients in the lower tertile were more likely to recur. For patients more than 1.11 standard deviations below the cut point, 37.5% failed versus 14.5% if they were above. This was significant on multivariate analysis along with PSA, Gleason score, and margin status, but there was no evaluation as to the actual effect on the prognostic ability of those factors.

### 3.21. Gene Arrays and Panels

With the use of gene expression micro arrays, the hope is that by screening a large number of genes, genes highly predictive of cancer recurrence could be identified. When using probe arrays, multiple genes can be identified that may predict for relapse. Several groups have evaluated this approach in predicting failure postprostatectomy. In a gene expression profile of 225 tumors with a median followup of 8 years [63], it was found that MUC1 was predictive of recurrence and AZGP1 was predictive of nonrecurrence. Both of these genes were predictive on multivariate analysis along with Gleason score, stage, and PSA. There were no actual outcome results given. A similar study [28] of 259 RRP patients with a median followup of 57 months searched also for markers using microarray assay. They found that the combination of EZH2 increased and ECAD decreased was most predictive of 5-year recurrence (38% versus 15% for those without that combination). On multivariate analysis, this ratio was significant along with PSA, margin status, and pathological stage, but not Gleason score. For organ-confined patients that were margin negative, those that were EZH2/ECAD elevated had a 27% recurrence rate, versus 10% for those that had a decreased ratio. They did not report on higher-risk patients. In a different study of 100 lymph node negative prostatectomy patients [65], with a median followup of 70 months an expression analysis of 12,625 transcripts identified 218 genes that were either up- or down regulated. Recurrence was defined as three rising PSA levels. The combination that predicted recurrence was deemed “poor markers”. For Gleason 6-7 cancers, the 5-year disease-free survival was 69%, but was 77% in the good marker group and 47% in the poor marker group. In Gleason 8-9 cancers, the 5-year disease-free survival rate was 26%, but was 67% with good markers and 0 with poor markers. On multivariate analysis, Gleason score and the gene expression markers were predictive of recurrence, but PSA and age were not. Using a postoperative nomogram [66] they identified poor risk patients by nomogram (undefined) who had a 28% 5-year disease-free survival, increasing 50% with good gene markers, but 19% with poor markers. In the nomogram predicted favorable group, 5-year disease-free survival was 81%, which was 87% with good markers and 59% with poor markers. The major limitation of the study is that there were only 21 patients in the training set and 79 patients in the validation set. 

 Another approach is to pool multiple genes in order to try to produce a more powerful predictive model. This has been successful in breast cancer [67, 68]. With that approach [69], using a 70 gene set in it was possible to predict 27/29 “aggressive” and 27/32 “nonaggressive” cancers and predicted 16 of the 18 failures. Unfortunately, it appears only 61 patients were evaluated; there was no indication of how the findings related to standard prognostic factors. In a more comprehensive study [70] of 639 patients selected for systemic recurrence, biochemical (PSA) recurrence and nonrecurrence at 7 years, the groups were evaluated for genes that differed between them. Patients with adjuvant treatment were not excluded and failure was with a PSA > 0.2 and rising. The patients were divided into training and a validation set. Ultimately, a 17-gene panel was determined to be predictive. Clinical models based on Gleason score, and pathological stage (PSA and age were not informative) demonstrated a correlation (area under the curve) of 0.76 (0.74–0.78), while the probe set was 0.85 and the combination of the two was 0.87. They reported that the AUC for the validation set was lower. They compared their results to those of other gene array studies and found that all the other models performed better than the clinical model alone (0.74, ranging from 0.76–0.86), with their 17 gene probe being the highest. All the validation sets were lower than the training sets for these genes. In an exploratory study [71] of 72 prostatectomy patients with a median followup of 28 months, 24% relapsed. After scanning for 59,619 probe sets, over 200 genes could be identified that are associated either positively with relapse. In another exploratory study [72], tissue from 37 failing patients and 42 nonfailing patients was tested with a 22,283-gene probe microarray. The first goal was to see if the identified genes (ultimately 5–8 were used) could correctly identify the failing versus the nonfailing patients, which it did 75% of the time. When combined into a nomogram, the predictive rate increased to 89%. Given that nomograms are the most robust incorporation of the standard prognostic factors; this would represent an example of how molecular data can increase the ultimate ability to predict who will fail. Unfortunately, the number of patients evaluated was very small, so any conclusions are tentative at best. One last study took a different approach. Rather than do a blind probe for over- or under expressed genes, they [73] evaluated a pre-existing class of predictive genes like those successful in breast cancer [74]. Although the actual genes are variable, most of the predictive breast cancer genes fall under the general classification of cell cycle progression genes. In evaluation of that class of genes in a large prostatectomy cohort [73] (442 with tissue, median followup 9.5 years), a panel of 31 was tested for their ability to predict recurrence. Overall, 10-year progression-free survival was 64%. When evaluated for the standard findings of PSA, Gleason score, and pathologic findings, the patients could be divided into two groups based on these clinical factors. The low-risk group were patients with Gleason < 7, organ-confined disease, and PSA < 10 ng/mL (actually, PSA up to 30 ng/mL did not change the risk). Their 10-year risk of biochemical failure (PSA > 0.1 ng/mL) was 17%, but for those with a low CCP score, it was 4% and for a high CCP score it was 24%. For clinical high-risk patients (Gleason ≥ 7 and/or nonorgan confined and/or PSA > 30), 10-year biochemical failure was 61%, which was 51% for low CCP score, and 64% for high score. On multivariate analysis, they the CCP score was predictive of recurrence.

 It is interesting to note, as pointed out previously [71], using multigene predictive models, there is little overlap in the genes that are found to be significant in each of the models. This is postulated to be a factor of a large number of genes and a high signal-to-noise ratio associated with the prediction of biochemical recurrence. The challenge then is to determine which of these are true prognostic markers and which are otherwise just testing anomalies. It will take large comprehensive studies to determine this.

## 4. Conclusion

 This paper covered those tissue markers that have been evaluated as prognostic factors in radical prostatectomy patients. These markers and multiple others have also been evaluated in patients with noncurative treatment and metastatic disease, as well as numerous tissue culture systems. There are undoubtedly many useful makers that will be identified, especially with the high volume analyses possible with the microarrays. At this time, none of them have been overwhelming in their prognostic ability nor do they have a value that mandates clinical use. 

The reason for the failure of molecular markers in consistently predicting outcome may partially be due to the variability between studies due to their methodological differences. Unfortunately, most studies are too small to comprehensively evaluate their ability to improve on the prognostic ability of the standard factors of PSA, Gleason score, and stage. Until that occurs, they will remain research curiosities.

 In terms of the pathway forward for a useful marker or signature in prostate cancer, we have many challenges. Our current classification of prostate cancer even at the very rudimentary molecular level is lacking. The estrogen, progesterone, and Her 2-neu receptor status of breast cancer has allowed stratification of a complex disease for clinical trials and as a paradigm for molecular signature generation. To date, this has not been possible in prostate cancer, although recent work suggests the imprinting of the TMPRSS2-ERG, PTEN, and androgen receptor configurational status may be suitable. Similarly, basic molecular predictors of outcome in the adjuvant, hormone-naïve, and castrate-resistant settings have been slow to develop in a disease that in its most aggressive form evolves over a decade. Finally, predictors of response to standard therapies have been difficult to characterize in the absence of a single dominant gene or the ability to subsegment the disease. To move forward, markers or gene signatures will need to have strong biological base, be linked to a therapeutic intervention and have enough strength to add to the formidable triad of stage, Gleason score, and serum PSA in prostate cancer.

## Figures and Tables

**Table 1:** Ki-67 and outcomes after radical prostatectomy. The table indicates whether Ki-67 was positive on univariate or multivariate analysis for predicting failure. The failure of the entire cohort is given and then the outcomes for patients where Ki-67 was elevated versus not elevated.

Study	#pts	Med (mean) months f/u	Include LN+ (#)	Include Adj RX (#)	Definition of failure^@^	Univariate positive	Multivariate positive	Group overall failure	Marker elevated outcome	Marker not elevated outcome
Khatami et al. [6]	50	(63)	No	NR	PSA > 0.2 × 2	Yes	NR	18%	NR	NR
Bubendorf et al. [7]	137	(64)	Yes (34)	Yes (60)	PSA, PAP, or ALP elevated*	Yes	Yes	29%	65% 5-yr dfs	78% 5-yr dfs
May et al. [8]	528	46 (49)	Yes (38)	No	PSA > 0.2	Yes	Yes	19% 5-yr dfs 78%	70% 5-yr dfs	88% 5-yr dfs
Miyake et al. [9]	193	63	Yes (13)	No	PSA > 0.2	Yes	Yes	21% 5-yr dfs 79%	A: 79% recur B: 20% recur	C: 4% recur
Rubio et al. [10]	91	46.5	NR	No	PSA ≥0.2	Yes	Yes	32%	42% 5-yr dfs	84% 5-yr dfs
Laitinen et al. [11]	229	66 (62)	Yes (NR)	Yes (4)	PSA ≥ 0.5 × 2	Yes	Yes	63% 5-yr dfs 10-yr 41%	5/10-yr dfs 2–15% : 62%/38% 16+% : 42%/27%	5/10-yr dfs 0-1% : 92%/65%
Moul et al. [4]	162	(54)	Yes (1)	NR	PSA > 0.2 × 2	Yes	No	38%	31% 6-yr dfs	72% 6-yr dfs
Bettencort et al. [12] (same patients as [4] above)	180	(53)	Yes (1)	NR	PSA > 0.2 × 2	Yes	No	60% 5-yr dfs	5-yr dfs 1+ 69% 2–4+ 44%	5-yr dfs 83%
Vis et al. [15]	112	113	Yes (6)	No	Clinical only^@^	Yes for clinical recurrence	No		Clinical dfs 5-yr 52% 10-yr 42%	Clinical dfs 5-yr 75% 10-yr 75%

NR: not reported.

^@^Most studies include biopsy-proven local recurrence and radiographic distant metastasis as failure in addition to PSA.

*Three factors: Ki-67, SV+, margin+; A = 2-3 factors, B = one factor, C = all 3 negative.

**Table 2:** p53, bcl-2, and outcomes after radical prostatectomy. The table indicates whether p53 and bcl-2 was positive on univariate or multivariate analysis for predicting failure. The failure of the entire cohort is given and then the outcomes for patients where the marker was elevated versus not elevated.

Study	#pts	Med (mean) months f/u	Include LN+ (*n*)	Include Adj RX (*n*)	Definition of failure^@^	Univariate positive	Multivariate positive	Group overall failure	Marker elevated outcome	Marker not elevated outcome
*P53*										
Theodorescu et al. [16]	71	127	Yes (1)	No	Clinical, PSA > 0.2	Yes	Yes	51% failed	15-yr cause-specific 38%	15-yr cause-specific 87%
Kuczyk et al. [17]	76	50	Yes (6)	No	Clinical	Yes	Yes	32% failed 20% died ca	33% died ca	16% died ca
Quinn et al. [18]	263	(56)	Yes (5)	Yes (99)	PSA ≥ 0.4 × 2	Yes	Yes	39% failed	32% 5-yr dfs	83% 5-yr dfs
Moul et al. [4]	162	(54)	Yes (1)	NR	PSA > 0.2 × 2	Yes	Yes	38%	39% 6-yr dfs	76% 6-yr dfs
Bauer et al. [19] same patients as [4]	175	(55)	Yes (1)	NR	PSA > 0.2 × 2	Yes	Yes	38%	45% failed 5-yr dfs 49%	23% failed 5-yr dfs 78%
Brewster et al. [20]	76	(38)	NR	No	PSA ≥ 0.2 × 2	Yes	Yes	30%	41% failed	21% failed
Goto et al. [21]	119	40	NR	No	PSA > 0.2	No	No	13% failed	40% failed	10% failed
Miyake et al. [9]	193	63	Yes (13)	No	PSA > 0.2	Yes	No	21% failed 5-yr dfs 79%	NR	NR
Wu et al. [23]	70	36.5	NR	NR	PSA > 0.2 × 2	No	No	30%	44% failed	26% failed
Osman et al. [24]	86	65	NR	Yes (33)	3 × PSA increase	NR	Yes	NR	0 5-yr dfs	68% 5-yr dfs

*BCL-2*										
Bauer et al. [19]	175	(55)	Yes (1)	NR	PSA > 0.2 × 2	Yes	Yes	38% failed	57% failed 5-yr dfs 33%	31% failed 5-yr dfs 69%
								38% failed	BCL2+ P53+ 5-yr dfs 25%	BCL2− P53− 5-yr dfs 80%
Brewster et al. [20]	76	(38)	NR	No	PSA > 0.2 × 2	Yes	Yes	30%	53% failed	24% failed
Goto et al. [21]	119	40	NR	No	PSA > 0.2	No	No	13% failed	21% failed	10% failed
Bubendorf et al. [22]	137	(64)	Yes (34)	Yes (60)	PSA, PAP, ALKP	NR	No	19% failed 5 yr dfs 78%	10-yr dfs 18%	10-yr dfs 52%
Miyake et al. [9]	193	63	Yes (13)	No	PSA > 0.2	No	No	21% failed	NR	NR
Wu et al. [23]	70	36.5	NR	NR	PSA > 0.2 × 2	Yes	Yes	30%	67% failed	28% failed

NR: not reported.

Most studies include clinical failure: biopsy-proven local recurrence and/or radiographic distant metastasis in addition to PSA.
